# A comparative analysis of the impact of repeated administration of flavan 3-ol on brown, subcutaneous, and visceral adipose tissue

**DOI:** 10.1515/med-2025-1152

**Published:** 2025-03-18

**Authors:** Naomi Osakabe, Hitomi Nakamura, Yamato Yoshida, Sae Katsuragawa, Naoki Iida, Yasuyuki Fujii, Ursula M. Jacob, Tilman Fritsch, Ali Abdelhameed, Vittorio Calabrese

**Affiliations:** Systems Engineering and Science, Graduate School of Engineering and Science, Shibaura Institute of Technology, Saitama, 337-8570, Japan; Department of Bioscience and Engineering, Faculty of System Science and Engineering, Shibaura Institute of Technology, 307 Fukasaku, Minumaku, Saitama, 337-8570, Japan; Healthcare AG, Zurich, Switzerland; NAM Institute, Salzburg, Austria; Department of Pharmaceutical Chemistry, College of Pharmacy, King Saud University, Riyadh, Saudi Arabia; Department of Biomedical and Biotechnological Sciences, University of Catania, Catania, Italy

**Keywords:** flavan 3-ols, brown adipose, inguinal adipose, epididymal adipose, sympathetic nerve

## Abstract

**Introduction:**

Flavan-3-ols (FLs), astringent polyphenols, are known to have low bioavailability and induce excessive sympathetic nervous system activation. This study aimed to compare the effects of FLs on brown, subcutaneous, and visceral adipose tissue in mice.

**Methods:**

C57BL/6J male mice fed a standard or high-fat diet were given water or 50 mg/kg FL orally by gavage for 2 weeks. Excised brown, inguinal, and epididymal fat tissues were prepared for frozen sectioning. After hematoxylin and eosin (HE) staining, the effects of FL administration on each adipose tissue were observed, and expression analysis of mitochondrial DNA genes was performed.

**Results:**

Repeated administration of FL had no morphological effects on brown adipose tissue or visceral fat. However, FL significantly reduced the cell size in subcutaneous fat and induced the appearance of multilocular structures. Furthermore, FL increased cytochrome B expression in subcutaneous fat. The results showed that FLs induce browning of subcutaneous fat in mice.

**Conclusion:**

This study showed that FL-induced enhancement of sympathetic nerve activity increased mitochondria in subcutaneous fat and promoted browning. However, no changes were observed in other adipose tissues. Further long-term administration is required to analyze the effects of FLs on adipose tissue thoroughly.

## 1 Introduction

Flavan-3-ols (FLs) are a fraction containing catechins, and their polymers are known to have a strong astringent taste. A recent large-scale long-term intake study of 21,000 older adults over 3.6 years reported that FL fractions significantly reduced deaths related to cardiovascular disease [1]. In addition, it has been known that FL exerts a significant impact on the metabolic system. This is evidenced by the observation that it markedly reduces plasma LDL, elevates HDL, and enhances glucose tolerance in intervention trials, lowering the risk factors associated with cardiovascular disease [2,3].

Although a small portion of ingested catechins is absorbed in the digestive tract, they are metabolized in intestinal epithelial cells or the liver so that almost no unchanged form is distributed in blood or tissues [3]. Furthermore, oligomeric procyanidins are hardly absorbed in the body [4]. Therefore, almost all ingested FLs are transported to the large intestine and excreted [2,3]. Therefore, the mechanism of action of FLs on these metabolic systems remains to be determined.

Conversely, it has been suggested that FLs may affect the metabolism of adipose tissue. In our previous report, a few hours after a single oral administration of FLs to mice, energy consumption, expression of uncoupling protein (UCP)-1 mRNA, a thermogenic protein in brown adipose tissue (BAT), and blood catecholamine concentrations significantly increased [5] and these changes were eliminated by β3-adrenaline antagonist [6]. These results indicate that FLs enhance sympathetic nervous activity.

It has been reported that sympathetic nerve stimulation, such as cold stress, promotes the browning of white adipocytes (WAT) or the differentiation of mesenchymal stem cells into beige adipose tissue. Like brown adipocytes, beige adipocytes are brown due to their high mitochondrial content and the presence of multilocular lipid droplets. In addition, the responses to sympathetic stimulation may vary among brown, beige, and white adipose tissue [7].

Accordingly, in this study, we aimed to conduct a pathological examination of the morphological alterations in adipocytes following the repeated administration of FLs and investigate the changes in mitochondrial content by gene expression analysis.

## 2 Materials and methods

### 2.1 Materials

FL from cocoa was prepared according to Natsume et al. [8]. The FL contained 4.56% (+)-catechin, 6.43% (−)-epicatechin, 3.93% procyanidin B2, 2.36% procyanidin C1, and 1.45% cinnamtannin A2. To determine a reference value, we also ascertained the polyphenol concentration in this fraction using the Prussian blue method, and it showed a value of 72.3%.

### 2.2 Animals and diets

C57BL/6J 12-week-old mice weighing 21–26 g, obtained from Charles River Laboratories Japan, Inc. (Tokyo, Japan) were used in this study.

### 2.3 Animal study

After being fed a basal diet for 14 days, mice were divided randomly into two feeding groups: a standard diet (MF^®^, Oriental Yeast Co. Ltd.Tokyo, Japan) or a high-fat diet (HFD32^®^, CLEA Japan Inc., Tokyo, Japan). The diet composition is shown in Table 1 and further divided into two groups: treated distilled water (DW) and 50 mg/kg FL (*n* = 8 each). At the end of the treatment period, all animals were sacrificed via decapitation by skilled researchers following the experimental procedures. Major organs and adipose tissues were collected. Brown, inguinal, and epididymal adipose tissues were prepared for frozen sectioning.

**Table 1:** Composition of diet using this study

		Normal diet	High-fat diet
		(MF^®^)	(HFD32^®^)
Water	g/100 g diet	6.2	8.1
Protein	g/100 g diet	25.5	23.2
Fat	g/100 g diet	32	4.9
Fiber	g/100 g diet	2.9	3.3
Mineral	g/100 g diet	4	5.9
Nitrogen-free matter	g/100 g diet	29.4	54.7
Energy	kcal/100 g diet	507.6	355.7

### 2.4 Histopathological observation

For histopathological observation, the brown (BAT), inguinal, and epididymal adipose tissues were blocked with FSC 22 Blue (3801481; Leica Biosystems, Nussloch, Germany), frozen with isopentane (Fuji Film Wako Chemical Corporation, Tokyo, Japan) on dry ice, and stored at −80°C. We cut 8 μm-thick slices of the adipose tissues to prepare frozen sections with a cryostat (CM1950; Leica Biosystems). Frozen sections were stained with HE according to standard methods. The study employed three pathological sections of the adipose tissue derived from a single animal. The sections were observed with a digital microscope utilizing the z-stack setting (BZ-X800; Keyence Corporation, Osaka, Japan). The analysis application BZ-H4A (Keyence Corporation) was employed. Three investigators blinded to the experimental groups and conditions performed histopathological observations of each adipose tissue.

### 2.5 Mitochondrial DNA expression analysis

Lysis buffer was added to each adipose tissue, and the tissue was homogenized. Neutral buffered phenol and chloroform were added to the supernatant, then stirred and centrifuged. Sodium acetate solution (3 M, pH 5.2) and cold 100% ethanol were added to the upper layer and stirred to extract the DNA. After air drying, DNA was dissolved in TE buffer and amplified by PCR using Luna Universal Probe qPCR Master Mix. The primer of *ND1* (Mm04225271_g1, ThermoFisher Scientific Inc., USA) and *CytB* (Mm04225274_g1, ThermoFisher Scientific Inc.), and β-actin (as an endogenous control, m02619580_g1, ThermoFisher Scientific Inc.) were used. The PCR conditions were initial denaturation at 95°C for 60 s, denaturation and extension at 95°C for 15 s, and 60°C for 60 s for 40 cycles. The ΔΔ*C*_t_ method was used for the analysis.

### 2.6 Statistical analysis

All data are reported as mean ± standard deviation. Statistical analyses were performed by two-way ANOVA followed by the Tukey, or non-parametric Wilcoxon, and Mann–Whitney U tests using GraphPad Prism. The *p* values calculated in the statistical tests are shown in Figure 4. The significance level was defined as *p* < 0.1, indicating a significant trend, and *p* < 0.05 indicating a significant difference.


**Ethical approval:** The study was approved by the Animal Care and Use Committee of the Shibaura Institute of Technology (Permit Number: AEA23008).

## 3 Results

A slight increase in body weight was observed after 2 weeks of ingesting a high-fat diet compared to a standard diet, but there was no difference between the DW and FL groups, as shown in Table 2. No differences were observed between the experimental groups in the weights of the heart, kidneys, liver, spleen, and adrenal gland. High-fat diet administration significantly increased the weights of the perirenal, epididymal, mesenteric, and inguinal adipose, but there was no difference between the DW and FL groups.

**Table 2:** Body and tissue weight of mice fed either normal or high-fat diet with distilled water or 50 mg/kg flavan 3-ols for 2 weeks

	Standard diet		High-fat diet	
	DW	FL	DW	FL
Body weight (initial, g)	23.6 ± 1.1	23.8 ± 1.5	23.9 ± 0.8	23.5 ± 0.7
Body weight (before dissection, g)	24.5 ± 0.8	25.1 ± 1.4	27.1 ± 1.6	26.8 ± 1.3
Body weight (final, g)	24.5 ± 0.9	25.1 ± 1.0	27.1 ± 1.4	26.8 ± 1.2
Heart (mg)	109.3 ± 9.3	111.7 ± 9.1	106.2 ± 5.9	104.1 ± 5.5
Kidney (mg)	261.5 ± 13.4	268.1 ± 21.7	263.3 ± 19.2	267.3 ± 16.9
Liver (mg)	949.4 ± 81.4	959.2 ± 29.2	932.8 ± 61.5	948.6 ± 65.4
Spleen (mg)	52.5 ± 11.5	56.6 ± 11.6	55.2 ± 7.7	49.6 ± 6.6
Adrenal grand (mg)	2.9 ± 0.6	3 ± 0.3	3.2 ± 0.3	3.0 ± 0.4
Perirenal adipose (mg)	116.2 ± 28.2	111.2 ± 37.5	308.0 ± 118.3***	309.8 ± 84.9***
Epididymal adipose (mg)	335 ± 35	319 ± 62.2	763.8 ± 233.4***	724.0 ± 179.2***
Mesenteric adipose (mg)	129.9 ± 30.8	135.9 ± 21.7	355.1 ± 159.5***	304.2 ± 68.7***
Inguinal adipose (mg)	17 ± 3.6	16.4 ± 5.2	33.7 ± 9.2***	37.1 ± 8.1***
Brown adipose (mg)	64.9 ± 11.2	57.6 ± 8.3	73.3 ± 16.2	72.3 ± 10.3

Values are mean ± SD (each group, *n* = 8). Statistical analyses were performed using two-way ANOVA followed by Tukey’s test. Significantly different from standard diet -DW: ***p < 0.0001.

**Figure 1:**
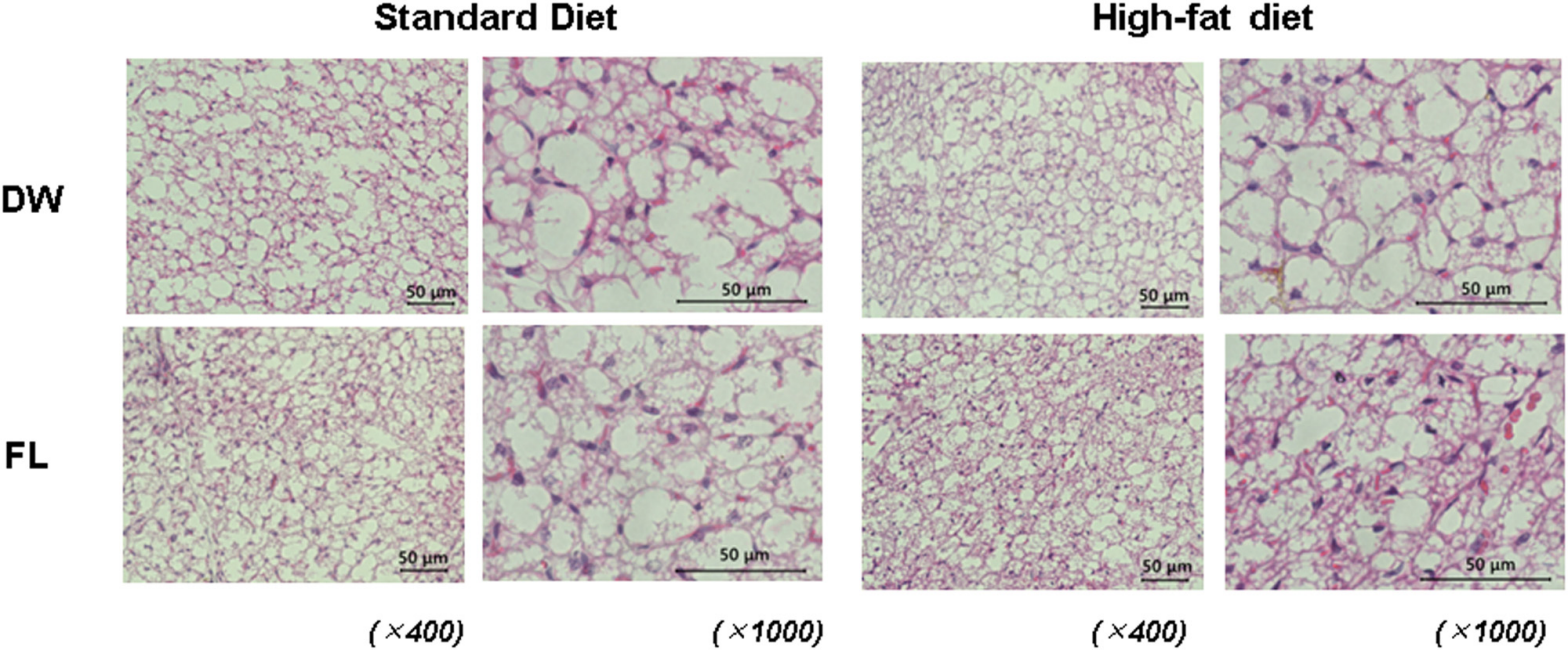
Histochemical image of brown adipose tissue (BAT) in mice either fed standard (left) or high-fat diet (right) after distilled water (DW) administration or 50 mg/kg flavan (FL) for 2 weeks. The upper panel shows the DW-administered group, while the lower panel illustrates the FL-administered group. Both images have been magnified 400× or 1,000×.

**Figure 2:**
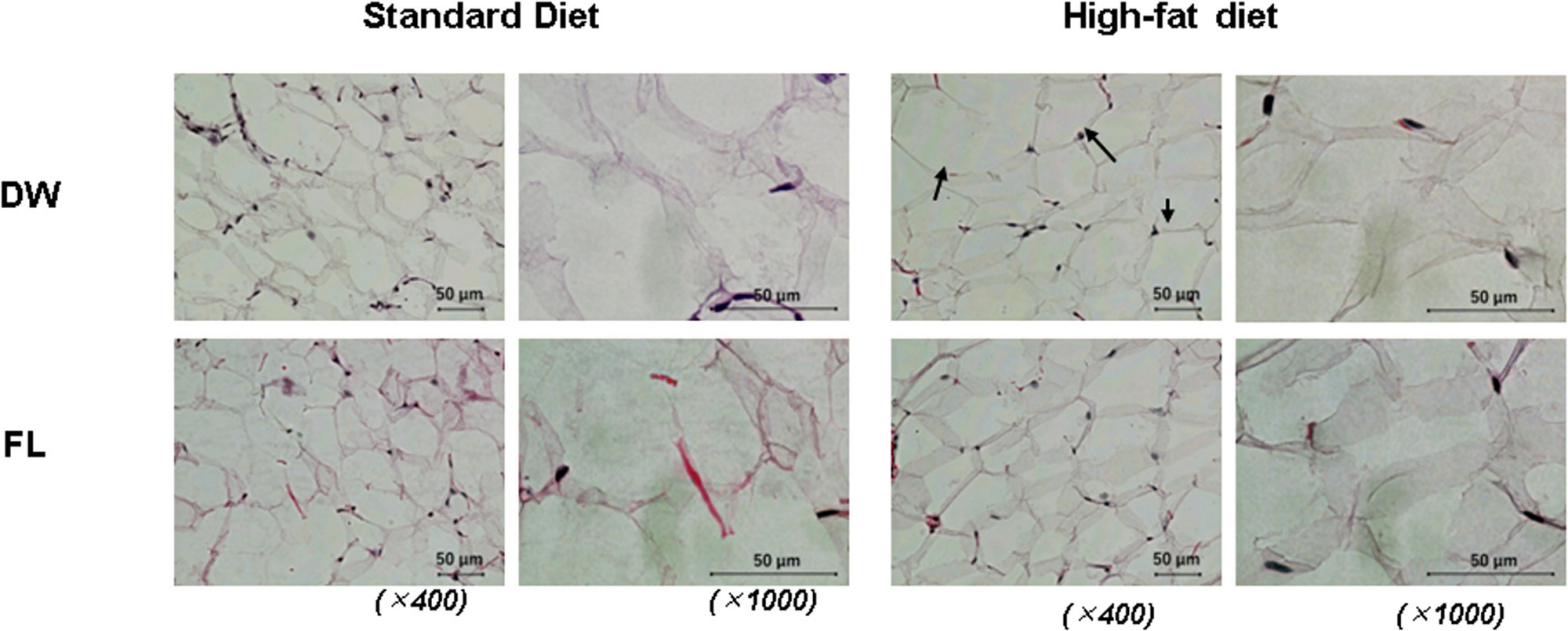
Histochemical image of visceral epididymal adipose (eWAT) in mice either fed standard (left) or high-fat diet (right) after distilled water (DW) administration or 50 mg/kgflavan 3-ols (FL) for 2 weeks. The upper panel shows the DW-administered group, while the lower panel illustrates the FL-administered group. Both images have been magnified 400× or 1,000×.

**Figure 3:**
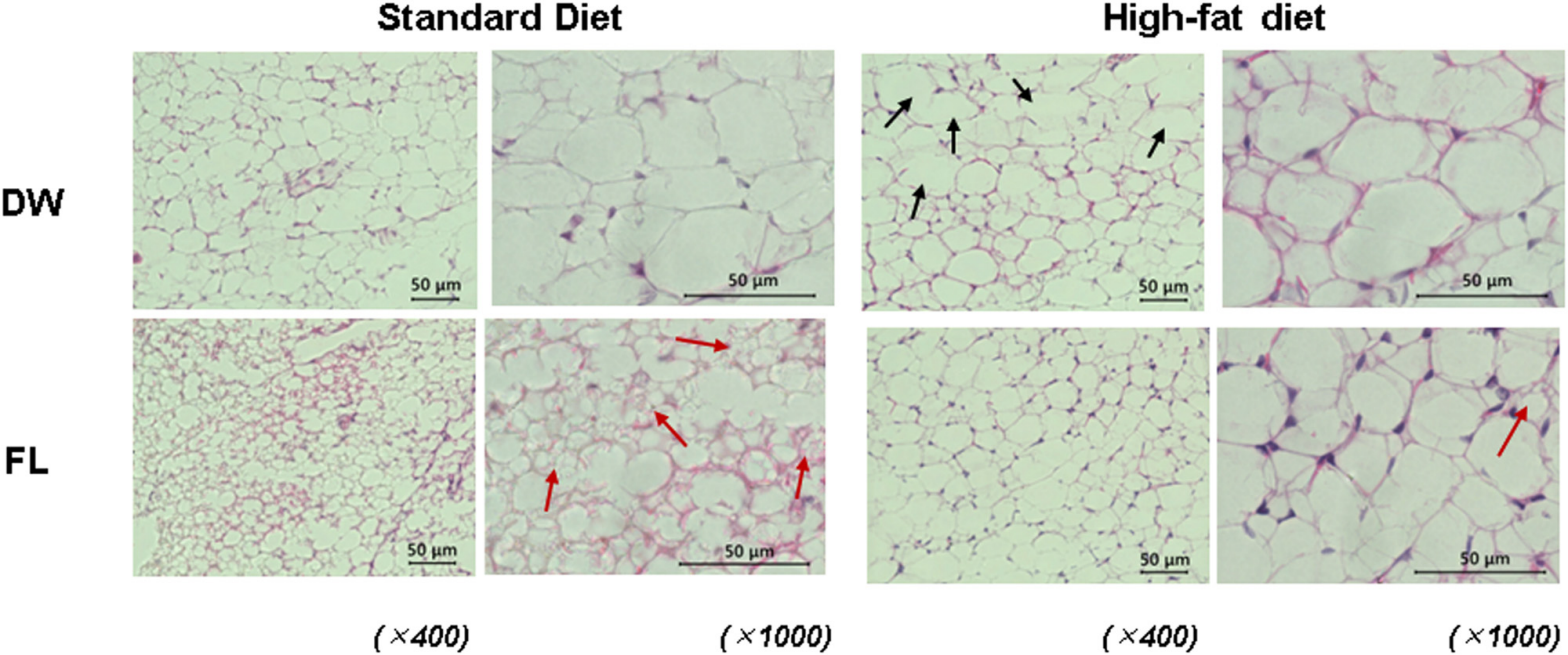
Histochemical image of subcutaneous inguinal adipose (iWAT) in mice either fed standard (left) or high-fat diet (right) after distilled water (DW) administration or 50 mg/kgflavan 3-ols (FL) for 2 weeks. The upper panel shows the DW-administered group, while the lower panel illustrates the FL-administered group. Both images have been magnified 400× or 1,000×.

Figures 1–3 show the typical HE-stained images of mice’s adipose tissues from each experimental group: standard diet – DW treatment group (a), standard diet – FL treatment group (b), high-fat diet – DW group (c), and high-fat diet – FL group (d). The images on the left are 400×, and the images on the right are 1,000×. Figure 1 shows the HE-staining images of brown adipose tissue from each mouse group. A 2-week high-fat diet feeding did not remarkably affect the morphology of brown fat, and no differences were found between the DW and FL groups. In the high-fat diet group, hypertrophied adipocytes were observed in the epididymal fat, as shown by the black arrows compared with the standard diet group. On the other hand, no such changes were observed in the FL group. HE-staining images of inguinal adipose tissue are shown in Figure 3. In the inguinal fat, enlarged adipocytes, as indicated by the black arrows, were observed in the high-fat diet group compared to the standard diet group. Conversely, repeated oral administration of FL caused a marked reduction in the cell size and the formation of multilocular lipid droplets, as indicated by the red arrows, in both standard and high-fat diets.

**Figure 4:**
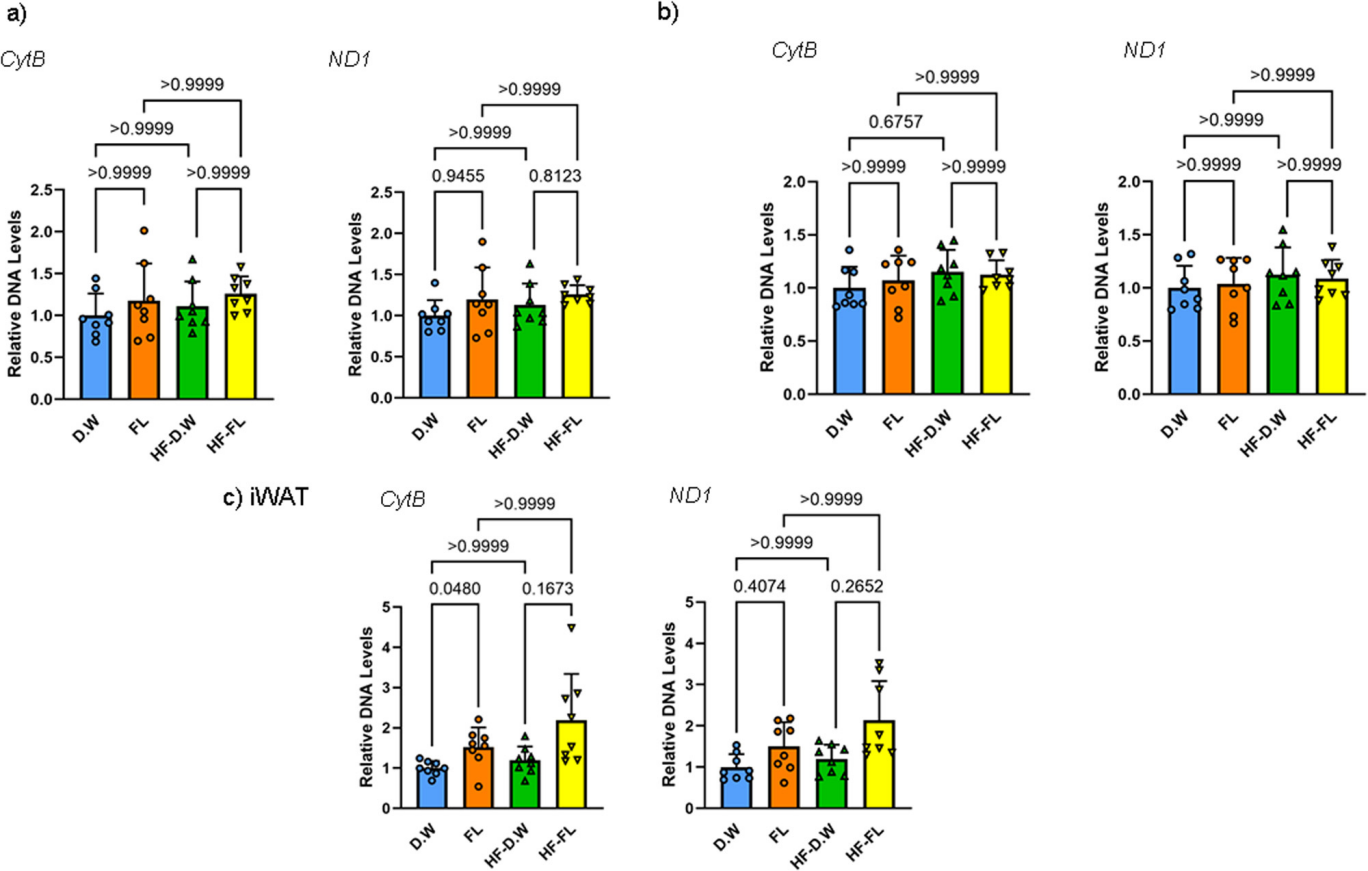
Mitochondrial DNA expression in brown (BAT, a), epididymal (eWAT, b), and inguinal adipose (iWAT, c) determined by RT-PCR. Each value represents mean ± standard deviation (*n* = 8, each). Statistical analyses were performed using non-parametric Wilcoxon and Mann*–*Whitney U tests. *Cyt B*, *cytochrome B.*

Figure 4 shows the levels of mitochondria DNA in mouse brown adipose tissue (a), epididymal adipose (b), and inguinal fat (c). Two weeks of a high-fat diet did not affect the expression of mitochondria DNA such as *ND1* and *CytB* in each adipose tissue (Figure 4a–c). FL did not change the expression of mitochondria DNA in brown adipose or epididymal adipose (Figure 4a and b). Conversely, FL administration significantly increased or tended to increase *ND1* in inguinal fat during standard and high-fat diets (Figure 4c).

## 4 Discussion

It has been reported that a single oral administration of FL enhanced the concentrations of noradrenaline in the blood [9] and urine [10], promoting circulation [11] and metabolic systems [5]. These changes were eliminated by co-administration of the adrenergic receptor blocker [6,12], suggesting that FL activates the sympathetic nervous system (SNS) [13].

Adipose tissue is known to be innervated only by sensory and sympathetic nerves [14] but not by parasympathetic or vagus nerves [15,16]. It is known that thermogenic UCP-1 is upregulated in response to noradrenergic input to beta β3 adrenalin receptors from the SNS, thereby promoting BAT energy expenditure. This observation was also replicated following a single administration of FL in our previous study [9]. Furthermore, repeated stimulation of the SNS has been observed to induce browning in white adipose tissue (WAT), forming discrete clusters of brown-like adipocytes, designated as beige adipocytes. These cells exhibit characteristics analogous to brown adipocytes, including multilocular accumulation of lipids, elevated mitochondrial content, and enhanced expression of UCP-1 [17]. In this study, hypertrophied adipocytes were detected in the eWAT of mice fed a high-fat diet compared to mice fed a standard diet, but such changes were minor in the FL-treated group (Figure 2). In iWAT, hypertrophied adipocytes were observed after high-fat diet intake as in WAT (Figure 3). However, a cell population with characteristics of beige cells was observed after FL administration in both mice fed a standard or high-fat diet (Figure 3). It has been reported that such adipose browning is determined by the expression level and sensitivity of the PR domain containing (Prdm) 16, a brown fat determinant [18]. The degree of browning susceptibility correlated with higher PRDM16 observed in subcutaneous iWAT compared with visceral eWAT of wild-type mice. Therefore, repeated sympathetic nerve stimulation-induced changes by FL were more pronounced in iWAT than in eWAT, with a particular prevalence of beige cells characterized by reduced cell size and multilocular lipid droplets.

Noradrenaline stimulation via the β3 adrenalin receptor activates adipocytes’ cAMP/protein kinase A (PKA) pathway, promoting triglyceride lipolysis. The cAMP/PKA pathway recruits p38 mitogen-activated protein (MAP) kinase and the mechanistic target of rapamycin complex (mTORC)1 pathway, which activates peroxisome proliferator-activated receptor γ coactivator (PGC)-1α [19]. PGC-1α is known to be more widely expressed in BAT than WAT, to upregulate UCP-1, and to increase the number of mitochondria with oxidative capacity, therefore being essential for thermogenesis [20]. Our previous report confirmed that the mRNA expression level of PGC-1α was significantly increased after a single oral administration of FL [6]. Therefore, an increase in PGC-1 expression, as shown in Figure 4c, induced by adrenaline stimulation, may be responsible for the increase in mitochondrial DNA observed in iWAT.

In this study, we investigated whether the early effects of FL intake on the stimulation of the SNS norepinephrine differed according to the type of fat, such as BAT, visceral fat, and subcutaneous fat. Therefore, the treatment period was set at 2 weeks. On the other hand, some studies have indicated that the administration of flavanols to mice for a period exceeding 8 weeks resulted in the formation of browning in visceral adipose and the activation of BAT, which has been associated with alterations in blood lipids, liver weight, and body weight [21]. A comprehensive analysis of the effects of FL on energy metabolism, including the browning of adipocytes, requires considering the role of the neuroendocrine system. Furthermore, the precise mechanism by which FL activates the SNS remains unclear. By elucidating this mechanism, it will become evident how FL intake contributes to maintaining homeostasis, including energy metabolism.

In conclusion, the sympathetic nervous activity induced by oral administration of FL for 2 weeks promoted the browning of iWAT. This effect was observed regardless of whether the rats were fed a standard or high-fat diet. The mechanism by which FL activates the SNS remains unclear and requires further elucidation through additional studies.
